# Effect of social mobility in family financial situation and housing tenure on mental health conditions among South Australian adults: results from a population health surveillance system, 2009 to 2011

**DOI:** 10.1186/s12889-015-2022-9

**Published:** 2015-07-17

**Authors:** Eleonora Dal Grande, Catherine R. Chittleborough, Jing Wu, Zumin Shi, Robert D. Goldney, Anne W. Taylor

**Affiliations:** Population Research and Outcome Studies, Discipline of Medicine, The University of Adelaide, SAHMRI, Level 7, North Terrace, 5005 Adelaide, Australia; School of Population Health, The University of Adelaide, Adelaide, Australia; Discipline of Psychiatry, The University of Adelaide, Adelaide, Australia

**Keywords:** Mental health, Socioeconomic factors, Social mobility, Life course epidemiology, Public health surveillance

## Abstract

**Background:**

To assess the association of socioeconomic position (SEP), measured by family financial situation and housing tenure in childhood and adulthood, with mental health conditions in adulthood.

**Methods:**

Representative cross-sectional population data were collected using a risk factor surveillance system in South Australia, Australia. Each month, a random sample were selected from the Electronic White Pages. Participants aged 25 years and above (n = 10429) were asked about doctor diagnosed anxiety, stress or depression, suicidal ideation, psychological distress, demographic and socioeconomic factors using Computer Assisted Telephone Interviewing (CATI). Social mobility measures were derived from housing status and perceived financial situation during adulthood and at 10 years of age.

**Results:**

The prevalence of psychological distress was 8.1 %, current diagnosed mental health condition was 14.8 % and suicidal ideation was 4.3 %. Upward mobility in family financial situation and housing tenure was experienced by 28.6 % and 19.3 %, of respondents respectively. Downward mobility was experienced by 9.4 % for housing tenure and 11.3 % for family financial situation. In the multivariable analysis, after adjusting for age, sex, childhood family structure and adult education, downward social mobility and stable low SEP (both childhood and adulthood), in terms of both housing tenure and financial situation, were positively associated with all three mental health conditions.

**Conclusion:**

People with low SEP in adulthood had poor mental health outcomes regardless of their socioeconomic circumstances in childhood. Policies to improve SEP have the potential to reduce mental health conditions in the population.

## Background

Mental health disorders present a significant public health burden and an estimated cost of over $6 billion per annum in Australia [1]. The lifetime prevalence of mental health disorders among Australians aged 16 to 85 years was 45 % in 2007, and one in five experience more than one mental disorder over a 12 month period [2]. Mental health disorders are a leading cause of disability burden in Australia, accounting for an estimated 24 % of the total years lost due to disability [3].

Socioeconomic and psychosocial environments have been associated with mental health [2, 4–6]. Cross-sectional studies in Australia have shown that current socioeconomic circumstances (low educational attainment, low household income, unemployed or unable to work) are associated with poor mental health [2, 6] but they do not include childhood social or economic circumstances measures that may influence adult mental illness. Life course approaches are being used to explain the development of poor mental illness in adulthood by examining the “long term effects on later health or disease risk of physical or social exposures during gestation, childhood, adolescence, young adulthood and later adult life” [7]. International studies have examined the relationship between socioeconomic position (SEP) in childhood and adulthood and mental illness [5, 8–11]. There are studies that have found no relationship between childhood SEP and poor mental health in adulthood when SEP in adulthood is taken into account [12, 13] but other studies have found associations with childhood SEP [11, 14, 15].

Several conceptual models have been developed to explain the effects of SEP over the life course on the development of health; one being the “mobility in the social structure of society” or social mobility [16, 17]. Simply, (intergenerational) social mobility refers to the change in SEP between generations: those who are disadvantaged in early life, based on the parents’ SEP, and remain disadvantaged through adulthood; those who are disadvantaged in early life but advantaged during adulthood (upward mobility); those who experience downward mobility from childhood to adulthood; and those who are advantaged during both early life and adulthood. It has been suggested that SEP in childhood can have a lasting effect on mental health in adulthood and people in different social mobility groups over the life course have varying health outcomes, with the downwardly mobile having poorer health, compared to those who maintained a high level of SEP across early life and adulthood [17, 18]. Many indicators have been used to measure early life SEP to ascertain social mobility: parental or family “material circumstances”; that is, the homes and neighbourhoods in which they can afford to live and by the living standards their income secures, such as occupational status, household income, and housing tenure [5, 19–21]; parental education since it is a mechanism to improve SEP [9, 20, 22]; and factors such as perceived job demands, job insecurity, perceived financial hardship, access to a car, healthier foods or computers, which are not limited to the basic material conditions [14, 21]. Some studies have examined how social mobility according to occupation or education of parent (s) and the individual in adulthood [8, 11, 12] is related to mental health in adulthood. Luo *et al.* [12] found that respondents who were upwardly socially mobile between childhood and adulthood had better mental health outcomes than respondents experiencing socioeconomic disadvantage across childhood and adulthood. They concluded that the impact of low SEP in childhood can be ameliorated if people experiencing low childhood SEP achieve higher status in adulthood.

The use of data from a representative population health surveillance system provides a large sample size to enable us to investigate differences in health and illness between different population groups and for monitoring the impact of policies and interventions aimed at reducing health inequities and intergenerational disadvantage over time [23, 24]. This paper aims to examine the association of three measure of mental health illness (psychological distress, self-reported diagnosed current mental illness, and suicidal ideation) among people who were socially mobile (upward or downward) between childhood and adulthood, with retrospectively recalled information about childhood family financial situation and housing tenure.

## Methods

### Sample

The study used data from the South Australian Monitoring and Surveillance System (SAMSS) collected between January 2009 and June 2011 for respondents aged 25 years and over. SAMSS, established in 2002, is a monthly telephone survey designed to systematically monitor the trends of health conditions, behavioural risk factors and other health services issues over time for the South Australian (SA) health system [25]. SAMSS utilises Computer Assisted Telephone Interview (CATI) method whereby a minimum of 600 randomly selected people of all ages each month are interviewed by trained health interviewers. All households in SA with a telephone connected and the telephone number listed in the telephone directory are eligible for selection. A letter introducing the survey is sent to the selected household and the person with the last birthday is chosen for interview. There are no replacements for non-responders. Up to ten call backs are made to the household to interview the selected persons. A total of 18430 interviews were conducted (64.7 % response rate) among respondents of all ages for the period January 2009 to June 2011. Ethical approval was obtained from SA Health and The University of Adelaide. All participants gave informed consent.

### Mental health outcomes

Psychological distress was determined using the Kessler 10 which are 10 questions based on the level of anxiety and depressive symptoms experienced in the previous four weeks at the time of the interview [26, 27]. The response categories are on a five point Likert scale ranging from ‘all of the time’ to ‘none of the time’, Each item was reverse scored (value 1 for ‘none of the time’ to 5 for ‘all of the time’ and summed to give scores ranging from 10 (no distress) to 50 (high risk of anxiety or a depressive disorder). Participants with low scores between 10 and 21 were deemed not to have psychological distress, while those with high scores between 22 and 50 were categorized as having psychological distress [2, 6]. Current self-reported diagnosed mental health condition was defined as being diagnosed by a doctor in the previous 12 months with anxiety, depression, a stress related problem or another mental health problem and/or currently receiving treatment for a mental health problem. Suicidal ideation was determined using four items drawn from the severe depression subscale of the 28-item General Health Questionnaire (GHQ-28) [28]. The suicidal questions were scored by applying the binary method to the four questions to produce a score ranging from 0 to 4 where a score of 1 or more indicated suicidal ideation [29]. Suicidal ideation is considered to be a necessary but not sufficient precursor to successful (or unsuccessful) suicide or suicide attempts including thoughts and preliminary actions [29].

### Socioeconomic position (SEP)

Previous work [23, 24] investigated a range of early-life SEP questions and found that questions on housing tenure and financial situation during childhood were suitable to include into a surveillance system like SAMSS. These questions complement existing questions on current SEP, and were found to have lower item non-response (1.7 % for housing tenure and 9.0 % for financial situation) compared to other SEP questions such as parental education (20.1 % for mother and 19.6 % for father) [24]. These early-life SEP questions were included from 2009. Respondents were asked about their housing tenure and financial situation currently and when they were 10 years old [30]. For family financial situation, high SEP was indicated by “being able to save a bit or a lot,” and low SEP was indicated by “not being able to save any money at all.” For housing tenure, living in a dwelling that the respondent or other family members owned or were purchasing indicated high SEP, and low SEP was indicated by living in a dwelling that was rented privately or from the government housing authority or having other housing circumstances. These two measures on social mobility according to financial situation and housing tenure were considered over other possible measures (parents’ education or occupational status) since they have been shown to have low item non-response and fewer differences between those who could and could not recall [24, 30]. Two social mobility variables were created from current and childhood financial situation and housing tenure. Each social mobility variable comprised four possible intergenerational trajectories: stable high, low in childhood and high in adulthood (upward mobility), high in childhood and low in adulthood (downward mobility), and stable low.

### Covariates

Demographic variables included age, gender and family structure at age 10. Other variables included highest level of education and the number of chronic conditions, which was derived from five co-morbidities: medically confirmed diabetes, current asthma, cardiovascular disease (heart attack, angina, heart disease and/or stroke), arthritis and osteoporosis. Health risk factor data included smoking status, body mass index (BMI) derived from self-reported weight and height and recoded into four categories (underweight, normal weight, overweight and obese) [31], and alcohol risk over a lifetime [32].

### Statistical analyses

There were 12567 respondents aged 25 years and over between January 2009 and June 2011. Analyses were conducted using respondents with complete data on mental illness, SEP and covariates (n = 10429). Missing items were mainly for respondents who could not answer or refused their financial situation at age 10 years (11.4 %), current financial situation (2.5 %) and dwelling status at age 10 years (2.4 %).

Analyses were conducted using SPSS Version 19 and Stata Version 12. Univariable analyses using chi-square tests compared the prevalence of each mental health condition across SEP demographic and health indicator variables. Associations between social mobility on family financial situation and housing tenure and each mental health condition were analysed using logistic regression models, adjusted for multiple covariates using SPSS. Model 1 adjusted for age and sex; family structure at age 10 was added in Model 2. Other variables including highest education level, chronic conditions, smoking, alcohol, and BMI were not adjusted for in these models as they were considered to be on the causal pathway between early life SEP and having a mental health condition in adulthood. The adjusted (age, sex, and family structure at age 10) predicted probability of having a mental health condition (psychological distress, self-reported mental health condition and suicidal ideation) by social mobility variables was calculated using logistic regression and presented using *marginsplot* syntax in Stata.

Data were weighted by area (metropolitan/rural), age, gender and probability of selection in the household to the most recent SA population data so that the results are representative of the SA population.

## Results

A description of the characteristics of respondents is shown in Table 1. Few differences existed between the response sample and analysis sample (table not shown). The prevalence of psychological distress was 8.1 % (95 % CI 7.6-8.7), current diagnosed mental health 14.8 % (95 % CI 14.1-15.5) and suicidal ideation was 4.3 % (95 % CI 3.9-4.7). The distribution of social mobility variables shows 28.6 % of respondents experienced upward mobility in family financial situation, and 19.3 % experienced upward mobility in housing tenure. Downward mobility was experienced by 11.3 % and 9.4 % for family financial situation and housing tenure, respectively.

**Table 1:** Prevalence and sample sizes for mental health outcomes, social mobility and sociodemographic variables, 25 years and over

	Analysis sample^a^ (n = 10429)	
	n	% (95 % CI)
MENTAL HEALTH OUTCOME		
Psychological distress	846	8.1 (7.6-8.7)
Mental health condition	1544	14.8 (14.1-15.5)
Suicidal ideation	450	4.3 (3.9-4.7)
SOCIAL MOBILITY		
Dwelling status		
High childhood and adulthood	6927	66.4 (65.5-67.3)
Low childhood, high adulthood (upward mobility)	2012	19.3 (18.5-20.1)
High childhood, low adulthood (downward mobility)	976	9.4 (8.8-9.9)
Low childhood and adulthood	515	4.9 (4.5-5.4)
Financial situation		
High childhood and adulthood	4727	45.3 (44.4-46.3)
Low childhood, high adulthood (upward mobility)	2979	28.6 (27.7-29.4)
High childhood, low adulthood (downward mobility)	1179	11.3 (10.7-11.9)
Low childhood and adulthood	1544	14.8 (14.1-15.5)
SOCIODEMOGRAPHIC VARIABLES		
Sex		
Male	5096	48.9 (47.9-49.8)
Female	5334	51.1 (50.2-52.1)
Age		
16 to 29 years	905	8.7 (8.2-9.2)
30 to 49 years	4357	41.8 (40.8-42.7)
50 to 69 years	3652	35.0 (34.1-35.9)
70 years and above	1516	14.5 (13.9-15.2)
Educational attainment		
No schooling to secondary	4779	45.8 (44.9-46.8)
Trade, certificate, diploma	2957	28.3 (27.5-29.2)
Degree or higher	2694	25.8 (25.0-26.7)
Dwelling status, adulthood		
Owned or being purchased	8939	85.7 (85.0-86.4)
Rented from the housing trust or government	377	3.6 (3.3-4.0)
Rented privately	868	8.3 (7.8-8.9)
Other	246	2.4 (2.1-2.7)
Financial situation, adulthood		
We spent more money than we got	345	3.3 (3.0-3.7)
We had just enough money to get through to the next pay	1853	17.8 (17.0-18.5)
Some money left over each week but we just spent it	525	5.0 (4.6-5.5)
We could save a bit every now and then	5743	55.1 (54.1-56.0)
We could save a lot	1963	18.8 (18.1-19.6)
Dwelling status, age 10		
Owned or being purchased	7903	75.8 (74.9-76.6)
Rented from the housing trust or government	1049	10.1 (9.5-10.7)
Rented privately	1028	9.9 (9.3-10.4)
Other	449	4.3 (3.9-4.7)
Financial situation, age 10		
We spent more money than we got	174	1.7 (1.4-1.9)
We had just enough money to get through to the next pay	4051	38.8 (37.9-39.8)
Some money left over each week but we just spent it	298	2.9 (2.6-3.2)
We could save a bit every now and then	4758	45.6 (44.7-46.6)
We could save a lot	1149	11.0 (10.4-11.6)
Family structure, age 10		
Family with child/children living with both biological or adoptive parents	9264	88.8 (88.2-89.4)
A step or blended family	267	2.6 (2.3-2.9)
Sole parent family	760	7.3 (6.8-7.8)
Other	133	1.3 (1.1-1.5)
HEALTH VARIABLES		
Current smoker	1617	15.5 (14.8-16.2)
Lifetime risk of alcohol-related harm	3222	30.9 (30.0-31.8)
Body mass index		
Overweight (> = 25 & <30)	3824	36.7 (35.7-37.6)
Obese (> = 30)	2403	23.0 (22.2-23.9)
Number of chronic conditions		
None	6252	59.9 (59.0-60.9)
1	2845	27.3 (26.4-28.1)
2	1014	9.7 (9.2-10.3)
3 to 5	319	3.1 (2.7-3.4)

^a^Analysis sample includes respondents with complete data on the relevant outcome and all social mobility variables and covariates

% prevalence estimate; CI confidence interval

Table 2 details the univariable associations between the three mental health conditions and social mobility, sociodemographic and health indicators. Those who had high SEP in both childhood and adulthood had the lowest prevalence of mental health conditions, and those with low SEP in both childhood and adulthood had the highest prevalence of mental health conditions. Respondents experiencing upward mobility had better mental health outcomes than those with low SEP in both childhood and adulthood. Mental health conditions were higher among respondents with lower educational attainment, and those who were current smokers, classified as obese, had chronic conditions, or had a step, blended family or sole parent family during childhood.

**Table 2:** Univariable analyses of mental health conditions by social mobility, socio-demographic and health-related variables (n = 10429), 25 years and over

	Psychological distress		Self-reported diagnosed mental health condition		Suicidal ideation	
	% (95 % CI)	*P* value	% (95 % CI)	*P* value	% (95 % CI)	*P* value
SOCIAL MOBILITY						
Dwelling status		<0.001		<0.001		<0.001
High childhood and adulthood	6.6 (6.0-7.2)		13.6 (12.8-14.4)		3.5 (3.1-4.0)	
Upward mobility	8.8 (7.7-10.2)		14.2 (12.7-15.8)		4.9 (4.1-6.0)	
Downward mobility	13.6 (11.5-15.8)		20.0 (17.6-22.6)		7.1 (5.6-8.9)	
Low childhood and adulthood	15.4 (12.6-18.8)		23.7 (20.2-27.6)		7.3 (5.3-9.9)	
Financial situation		<0.001		<0.001		<0.001
High childhood and adulthood	4.2 (3.6-4.8)		11.8 (10.9-12.7)		2.4 (2.0-2.9)	
Upward mobility	6.5 (5.6-7.4)		12.3 (11.1-13.5)		3.3 (2.7-4.0)	
Downward mobility	14.7 (12.8-16.9)		20.8 (18.6-23.3)		8.5 (7.0-10.2)	
Low childhood and adulthood	18.2 (16.4-20.3)		24.4 (22.3-26.6)		8.9 (7.6-10.5)	
SOCIODEMOGRAPHIC VARIABLES						
Sex		<0.001		<0.001		0.491
Male	6.6 (6.0-7.4)		12.5 (11.6-13.4)		4.5 (3.9-5.1)	
Female	9.5 (8.8-10.3)		17.0 (16.1-18.1)		4.2 (3.7-4.7)	
Age		0.004		<0.001		0.571
16 to 29 years	8.3 (6.7-10.3)		17.5 (15.2-20.2)		4.0 (2.9-5.4)	
30 to 49 years	8.9 (8.0-9.7)		14.9 (13.9-16.0)		4.6 (4.0-5.3)	
50 to 69 years	8.1 (7.3-9.0)		15.4 (14.2-16.6)		4.3 (3.7-5.0)	
70 years and above	5.9 (4.8-7.2)		11.5 (10.0-13.2)		3.8 (3.0-4.9)	
Educational attainment		<0.001		0.044		0.001
No schooling to secondary	9.1 (8.4-10.0)		15.6 (14.6-16.6)		4.9 (4.4-5.6)	
Trade, certificate, diploma	8.3 (7.3-9.3)		14.9 (13.6-16.2)		4.4 (3.7-5.2)	
Degree or higher	6.1 (5.3-7.1)		13.4 (12.2-14.8)		3.1 (2.5-3.8)	
Family structure, age 10		<0.001		<0.001		<0.001
Family with child/children living with both biological or adoptive parents	7.7 (7.2-8.3)		14.0 (13.3-14.7)		4.1 (3.7-4.5)	
A step or blended family	13.2 (9.6-17.7)		22.7 (18.1-28.1)		5.1 (3.0-8.4)	
Sole parent family	10.5 (8.5-12.9)		20.4 (17.7-23.4)		5.9 (4.4-7.8)	
Other	10.9 (6.7-17.3)		22.0 (15.8-29.7)		7.2 (3.9-12.9)	
HEALTH VARIABLES						
Smoking status		<0.001		<0.001		<0.001
Non/ex smoker	7.0 (6.4-7.5)		13.5 (12.8-14.2)		3.7 (3.3-4.1)	
Current smoker	14.4 (12.8-16.2)		21.9 (19.9-24.0)		7.6 (6.4-9.0)	
Lifetime risk of alcohol-related harm		<0.001		<0.001		<0.001
Does not drink	12.1 (10.7-13.6)		19.2 (17.4-21.0)		5.8 (4.8-6.9)	
No risk	7.0 (6.3-7.7)		12.6 (11.8-13.6)		3.4 (2.9-3.9)	
Lifetime risk of harm	7.7 (6.9-8.7)		15.9 (14.7-17.2)		5.0 (4.3-5.8)	
Body mass index		<0.001		<0.001		<0.001
Healthy (> = 18.5 & <25)	6.7 (5.9-7.5)		13.3 (12.3-14.5)		3.4 (2.8-4.0)	
Underweight (<18.5	12.3 (8.0-18.4)		11.7 (7.5-17.8)		5.9 (3.1-10.8)	
Overweight (> = 25 & <30)	6.2 (5.5-7.0)		12.6 (11.6-13.7)		3.9 (3.4-4.6)	
Obese (> = 30)	12.2 (10.9-13.5)		20.6 (19.0-22.2)		6.0 (5.1-7.0)	
Number of chronic conditions		<0.001		<0.001		<0.001
None	5.8 (5.2-6.4)		11.2 (10.4-12.0)		3.1 (2.7-3.6)	
1	9.1 (8.1-10.2)		17.9 (16.5-19.4)		5.3 (4.5-6.1)	
2	15.4 (13.3-17.7)		22.9 (20.4-25.6)		7.1 (5.7-8.8)	
3 to 5	22.1 (17.9-27.0)		32.6 (27.7-37.9)		10.2 (7.4-14.1)	
Overall	8.1 (7.6-8.7)		14.8 (14.1-15.5)		4.3 (3.9-4.7)	

% prevalence estimate; CI confidence interval

**Fig. 1:**
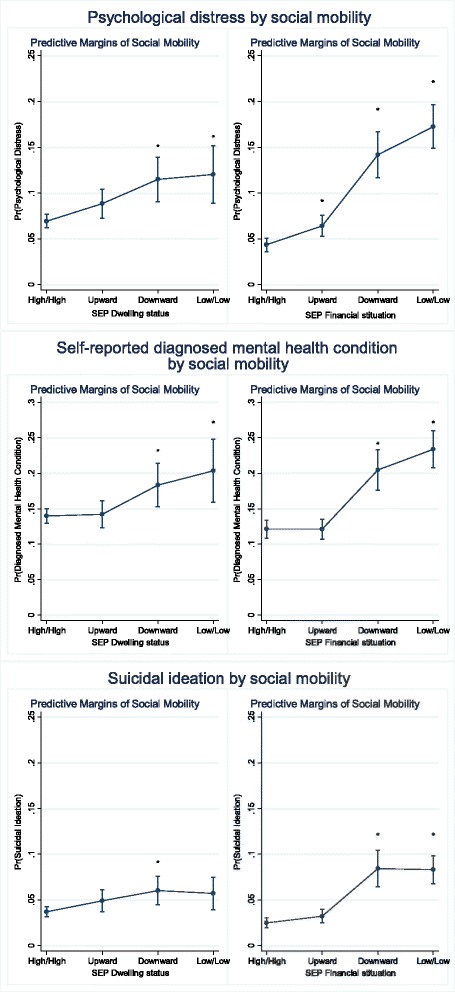
Adjusted probability of mental health illnesses (psychological distress, self-reported diagnosed mental health condition, and suicidal ideation) by social mobility (dwelling status and financial situation), aged 15 years and over. Note: Derived using the MARGINS command in Stata version 12.0 based on logistic regression models adjusted by age, sex, family structure at age 10; ‘*’ denotes social mobility category significantly different from stable high category (χ^2^ test, p<0.05) using contrast option in *Margins*

Using multivariable analysis (Table 3), after adjusting for age and sex, and childhood family structure (Model 2), downward social mobility between childhood and adulthood, in terms of both housing tenure and financial situation, was associated with each mental health condition. Respondents experiencing low SEP during both childhood and adulthood were also more likely to experience all three mental health conditions. Upwardly socially mobile respondents were no more likely to experience a diagnosed mental health condition than respondents experiencing high SEP during both childhood and adulthood. Similarly, these findings are reflected in Fig. 1, which shows the effects of each category of social mobility by computing the adjusted predicted probability of having a mental health illness (using the three measures) after adjusting for age, sex, and childhood family structure. The probability of having a mental health condition increases for people experiencing low SEP in adulthood regardless of SEP during childhood using family financial situation.

**Table 3:** Social mobility related to dwelling status and financial situation and risk of mental health conditions, 25 years and over

SEP at age 10	Adult SEP	Model 1 Adjusted age, sex		Model 2 + family structure at age 10	
		OR (95 % CI)	*P* value	OR (95 % CI)	*P* value
Psychological distress (K10)					
Dwelling status					
High	High	1.00		1.00	
Low	High (upward mobility)	1.33 (1.10-1.62)	0.003	1.31 (1.08-1.59)	0.006
High	Low (downward mobility)	1.80 (1.45-2.23)	<0.001	1.79 (1.44-2.22)	<0.001
Low	Low	1.94 (1.48-2.55)	<0.001	1.90 (1.44-2.50)	<0.001
Financial situation					
High	High	1.00		1.00	
Low	High (upward mobility)	1.53 (1.24-1.89)	<0.001	1.51 (1.22-1.87)	<0.001
High	Low (downward mobility)	3.69 (2.97-4.59)	<0.001	3.68 (2.96-4.58)	<0.001
Low	Low	4.71 (3.86-5.74)	<0.001	4.66 (3.81-5.69)	<0.001
Self-reported diagnosed mental health condition					
Dwelling status					
High	High	1.00		1.00	
Low	High (upward mobility)	1.06 (0.92-1.24)	0.422	1.02 (0.88-1.19)	0.784
High	Low (downward mobility)	1.42 (1.19-1.69)	<0.001	1.40 (1.17-1.67)	<0.001
Low	Low	1.72 (1.38-2.15)	<0.001	1.60 (1.27-2.00)	<0.001
Financial situation					
High	High	1.00		1.00	
Low	High (upward mobility)	1.04 (0.90-1.20)	0.618	1.00 (0.86-1.15)	0.951
High	Low (downward mobility)	1.88 (1.59-2.23)	<0.001	1.88 (1.59-2.22)	<0.001
Low	Low	2.31 (1.98-2.68)	<0.001	2.23 (1.92-2.60)	<0.001
Suicidal ideation					
Dwelling status					
High	High	1.00		1.00	
Low	High (upward mobility)	1.39 (1.08-1.79)	0.01	1.35 (1.05-1.74)	0.02
High	Low (downward mobility)	1.70 (1.28-2.26)	<0.001	1.69 (1.27-2.24)	<0.001
Low	Low	1.65 (1.14-2.39)	0.008	1.59 (1.09-2.31)	0.016
Financial situation					
High	High	1.00		1.00	
Low	High (upward mobility)	1.33 (1.01-1.77)	0.045	1.30 (0.98-1.73)	0.069
High	Low (downward mobility)	3.59 (2.72-4.75)	<0.001	3.60 (2.72-4.77)	<0.001
Low	Low	3.64 (2.79-4.73)	<0.001	3.55 (2.72-4.63)	<0.001

OR odds ratio; CI confidence interval

## Discussion

This study used representative cross-sectional population data from a surveillance system and demonstrated that people with low SEP in adulthood were more likely to experience poor mental health outcomes regardless of their family financial situation or housing tenure in childhood. Even when controlling for the effects of family structure at age 10 the association between low SEP in adulthood (downward mobility and stable low SEP) and mental health illnesses decreased by only 1 to 9 %.

These findings imply that policies to improve housing and providing financial support could have a positive influence on mental health outcomes in the population, to which there is no simple singular answer. To improve housing for both renters and owners is a complex undertaking which has been shaped over many decades through various interactions between legislation or regulation, government policies (state and federal), local and global market forces (for example the 2008 Global Financial Crisis) and family needs in terms of physical requirements and psychosocial needs such as privacy, safety and security [33]. Continual housing assistance programs including public housing and rent assistance for families has long-term benefits in terms of improved access to education, employment and income levels [34, 35]. Other studies have suggested better mechanism for first home buyers and policies to adapt to the ongoing risks (unemployment, reduced work hours, rise in interest rates, unexpected illness) faced by home owners with a mortgage who are financially stressed [33, 36]. Even though financial stress or the difficulties regarding housing tenure cannot be eliminated, programs to develop resilience and coping skills can be taught and possibly reduce mental illness later in life.

The association of downward social mobility with poorer mental health in this study is similar to a previous study of Norwegian men, where suicide and schizophrenia disability were associated with a combination of high parental and low own education level [11]. In a longitudinal study of older Americans, people experiencing upward social mobility had similar levels of depressive symptoms to those experiencing relatively high socioeconomic circumstances across the life course [12]. Cumulative effects of adult SEP on mental disorders were also not influenced by adjustment for childhood SEP in analyses of the 1958 British Birth Cohort [37]. Even taking into account education, which has been shown to influence SEP in adulthood [37, 38], poor mental health was more prevalent among those with low SEP in adulthood.

To our knowledge, this is the first paper of its kind in the Australian context and is unique in having a large sample on the general population so that the conclusions are likely to be generalizable to the Australian population. The prevalence of each mental health condition in this study was comparable to other Australian population estimates [2, 39–41]. The large sample size enable us to examine associations of SEP with suicidal ideation, an outcome of low prevalence. The design of this study is reliant on retrospective self-reported SEP data, and the memory of participants could result in over-or under-reporting of early life circumstances, with a high proportion unable to recall these circumstances. As a result, this could weaken the observed associations. The choice of these SEP variables at age 10 were based on previous studies that measured performance in recall; including how easily and accurately these circumstances were recalled, whether the indicators have the same meaning over time and for different population groups such as migrants and older people, and indicators that minimised socially desirable responses (for example embarrassment of early childhood circumstances) [13, 24, 42]. This study demonstrated that having missing responses for childhood housing tenure and family financial situation was associated with a few variables [24, 42], making recall suitable for inclusion in a surveillance system like SAMSS. However, these studies did not examine the accuracy of recall of childhood housing tenure and family financial. Other measures of childhood SEP, including parental education and occupation, have been shown to be less well recalled by adults [24] and are not included in this surveillance system. While 17 % of the response sample was excluded from analyses because of missing responses, few differences existed between the response and analysis samples. Missing data were mainly due to those who could not recall childhood circumstances: 2.2 % could not recall their housing situation and 11.4 % did not state or could not recall their family financial situation when they were 10 years of age. A small proportion (0.7 %) were excluded because their childhood circumstances were atypical, for example, in an orphanage, concentration camp, institution, displaced, refugee.

Another limitation of this study using self-reported data, is that the respondents may provide socially desirable responses, which may result in the under-reporting of having a mental health condition. Clinical assessment of mental health outcomes would be ideal but is costly and resource intensive, especially in a population surveillance system such as SAMSS. Using a mental health service or professional as an indicator is problematic as middle aged people are higher service users compared to the younger age group which has the highest prevalence [6]. Hence, our study is reliant on established, validated instruments such as Kessler 10 for psychological distress or GHQ-28 for suicidal ideation that are suitable for assessment via the telephone. Furthermore, due to the cross-sectional nature of the study, the mental health instruments focus on the most recent four week prior to the interview, or current treatment, therefore, they only measure current mental health status; not past mental health episodes. Again, this could result in under-estimation of the strength of the association. Our study was unable to adjust for factors (were not included in SAMSS) which have been shown to contribute to mental illnesses in adulthood. These include family history of mental illness, family relationships or social networks during childhood, childhood mental illness [43] and factors such violence, abuse, neglect, lack of family cohesion [38, 44]. Possibly, those with poor physical and mental health are more likely to experience downward social mobility or a barrier to upward mobility [45]. Possible bias from using only listed telephone numbers can be considered a weakness of this study. Although telephones are connected to a large number of Australian households, not all are listed (mobile-only households and silent numbers) whereby 9 % of South Australian households are mobile-only and 69 % of households do not have their landline or mobile number listed [46]. Previous work has shown that inclusion of unlisted landline numbers in the sample did not impact on the health estimates [47]. The proportion of mobile-only households have increased in South Australia to 21 % as of 2011 [48, 49] with a very small proportion (4 %) listed in the telephone directory [46]. The impact on health estimates by excluding this group from the sample in Australia is unknown to date. However, the characteristics of people living in mobile-only households have been found to be distinctly different. Hence, potential bias may exist in the estimates obtained in this study.

## Conclusion

Aside from the cross-sectional nature and limitations, this study has provided a useful, albeit simplified, foundation to the understanding of the relationship of SEP and mental health over the life course. The opportunity to reduce poor mental health in adulthood may be achieved by improving and providing continual support for housing affordability and security, and minimising financial stress hence decreasing socioeconomic disadvantages. With the recent economic situation, there is a possibility of more people experiencing downward social mobility and increased resources need to be allocated to prevent or treat mental health, hence, reducing the burden on health services. With a surveillance system like SAMSS, future analyses can monitor prevalence of mental health conditions in the population to determine if policies aimed at addressing mental health issues have been effective, in particular for specific social mobility subgroups.
